# pH and redox dual-responsive nanoparticles based on disulfide-containing poly(β-amino ester) for combining chemotherapy and COX-2 inhibitor to overcome drug resistance in breast cancer

**DOI:** 10.1186/s12951-019-0540-9

**Published:** 2019-10-17

**Authors:** Sipei Zhang, Nan Guo, Guoyun Wan, Tao Zhang, Chunyu Li, Yongfei Wang, Yinsong Wang, Yuanyuan Liu

**Affiliations:** 1 Tianjin Key Laboratory on Technologies Enabling Development of Clinical Therapeutics and Diagnostics (Theranostics), School of Pharmacy; Department of Genetics, School of Basic Medical Sciences; Department of Integrated Traditional Chinese and Western Medicine, International Medical School, Tianjin Medical University, Qixiangtai Road 22, Tianjin, 300070 China; 2 Choate Rosemary Hall, Class of 2019, Wallingford, CT 06492 USA

**Keywords:** pH and redox dual-responsive, Nanoparticle, Celecoxib, Drug resistance, Breast cancer

## Abstract

**Background:**

Multidrug resistance (MDR) generally leads to breast cancer treatment failure. The most common mechanism of MDR is the overexpression of ATP-binding cassette (ABC) efflux transporters such as P-glycoprotein (P-gp) that reduce the intracellular accumulation of various chemotherapeutic agents. Celecoxib (CXB), a selective COX-2 inhibitor, can dramatically enhance the cytotoxicity of doxorubicin (DOX) in breast cancer cells overexpressing P-gp. Thus it can be seen that the combination of DOX and CXB maybe obtain synergistic effects against breast cancer by overcoming drug resistance.

**Results:**

In this study, we designed a pH and redox dual-responsive nanocarrier system to combine synergistic effects of DOX and CXB against drug resistant breast cancer. This nanocarrier system denoted as HPPDC nanoparticles showed good in vitro stability and significantly accelerated drug releases under the acidic and redox conditions. In drug-resistant human breast cancer MCF-7/ADR cells, HPPDC nanoparticles significantly enhanced the cellular uptake of DOX through the endocytosis mediated by CD44/HA specific binding and the down-regulated P-gp expression induced by COX-2 inhibition, and thus notably increased the cytotoxicity and apoptosis-inducing activity of DOX. In MCF-7/ADR tumor-bearing nude mice, HPPDC nanoparticles showed excellent tumor-targeting ability, remarkably enhanced tumor chemosensitivity and reduced COX-2 and P-gp expressions in tumor tissues.

**Conclusion:**

All results demonstrated that HPPDC nanoparticles can efficiently overcome drug resistance in breast cancer both in vitro and in vivo by combining chemotherapy and COX-2 inhibitor. In a summary, HPPDC nanoparticles show a great potential for combination treatment of drug resistant breast cancer.

## Background

Breast cancer is a common type of cancer, and it is the most commonly diagnosed cancer and the leading cause of cancer death for females [1, 2]. Among various treatment strategies, chemotherapy has remained one of the most common tools for breast cancer treatment. Multidrug resistance (MDR), characterized by a simultaneous resistance to diverse chemotherapeutic drugs, is a major impediment towards chemotherapy fighting breast cancer [3, 4]. Several mechanisms have been reported to be responsible for MDR, including ATP-binding cassette (ABC) transporters, anti-apoptotic proteins, DNA repair enzymes, etc. [5, 6]. Thereinto, ABC transporters are a kind of transmembrane proteins which transport a wide variety of substrates across extra and intracellular membranes including chemotherapeutic drugs. P-glycoprotein (P-gp), encoded by MDR1, is a well-characterized ABC-transporter and found to be over-expressed or constitutively active in 50% of breast cancers [7–9]. Although much research has been devoted to exploring P-gp inhibitors for overcoming MDR, only limited success has been achieved in clinical practice [10].

Cyclooxygenase 2 (COX-2) is an inducible form of the enzyme that catalyzes the first step in prostanoid synthesis. Many investigations have reported that COX-2 plays an important role in cancer development and progression through multiple mechanisms e.g. promoting cell division, inhibiting cell apoptosis, altering cell adhesion, and stimulating tumor neovascularization, so that it can be used as a molecular target for cancer treatment [11, 12]. COX-2 is also found to be involved in MDR through up-regulating efflux transporters (e.g. P-gp etc.), which minimize intracellular drug concentration [13, 14]. The direct evidence is showing that COX-2 inhibitors can specifically increase the chemosensitivity of cancer cells overexpressing P-gp. Celecoxib (CXB), a selective COX-2 inhibitor, can effectively prevent the development of chemoresistance in breast cancer cells induced by doxorubicin (DOX) by suppressing P-gp expression and function, and furthermore synergistically boost the cytotoxicity of DOX in these drug-resistant cells [15–18]. Thus it can be seen that the combination of DOX and CXB maybe obtain synergistic effects against breast cancer by overcoming drug resistance. However, CXB is water-insoluble and has very low bioavailability, and also DOX combined with CXB perhaps produce some unexpected side effects due to their lack of tumor-targeting specificity. All these will dramatically limit their clinical applications in breast cancer treatment.

Polymeric nanoparticles, with nontoxicity in vivo, can not only greatly improve the solubility of insoluble drugs, but also targetedly deliver drugs to the tumor site through the permeability and retention (EPR) effect and the surface-modification with ligands or antibodies [19, 20]. To some extent, polymeric nanoparticles can overcome drug resistance in cancers by intracellularly delivering chemotherapeutic drugs through specific cellular internalization and/or directly interacting with efflux pumps [21]. Moreover, the nanoparticles incorporated with stimuli-responsive properties can controllably release anticancer drugs at the target site by responding to the internal stimuli (e.g., pH, temperature, enzyme and redox) and/or the external stimuli (e.g., magnetic, light and ultrasound) [22]. Consideration of the acidic pH values of extracellular milieu and endocytic-related organelles and the high cellular content of glutathione (GSH) in solid tumors, pH- and/or redox-responsive nanoparticles will help to chemotherapeutic drugs to fully exert antitumor effects and meanwhile alleviate their toxicity on normal tissues [23–25]. Most importantly, polymeric nanoparticles usually have a large capacity for drug loading and can efficiently encapsulate multiple anticancer drugs with different mechanisms, which is suitable for cancer combination treatment [26, 27]. Thus well-designed intelligent polymeric nanoparticles are expected to solve the problems described above in combination of DOX and CXB in breast cancer treatment.

**Fig. 1:**
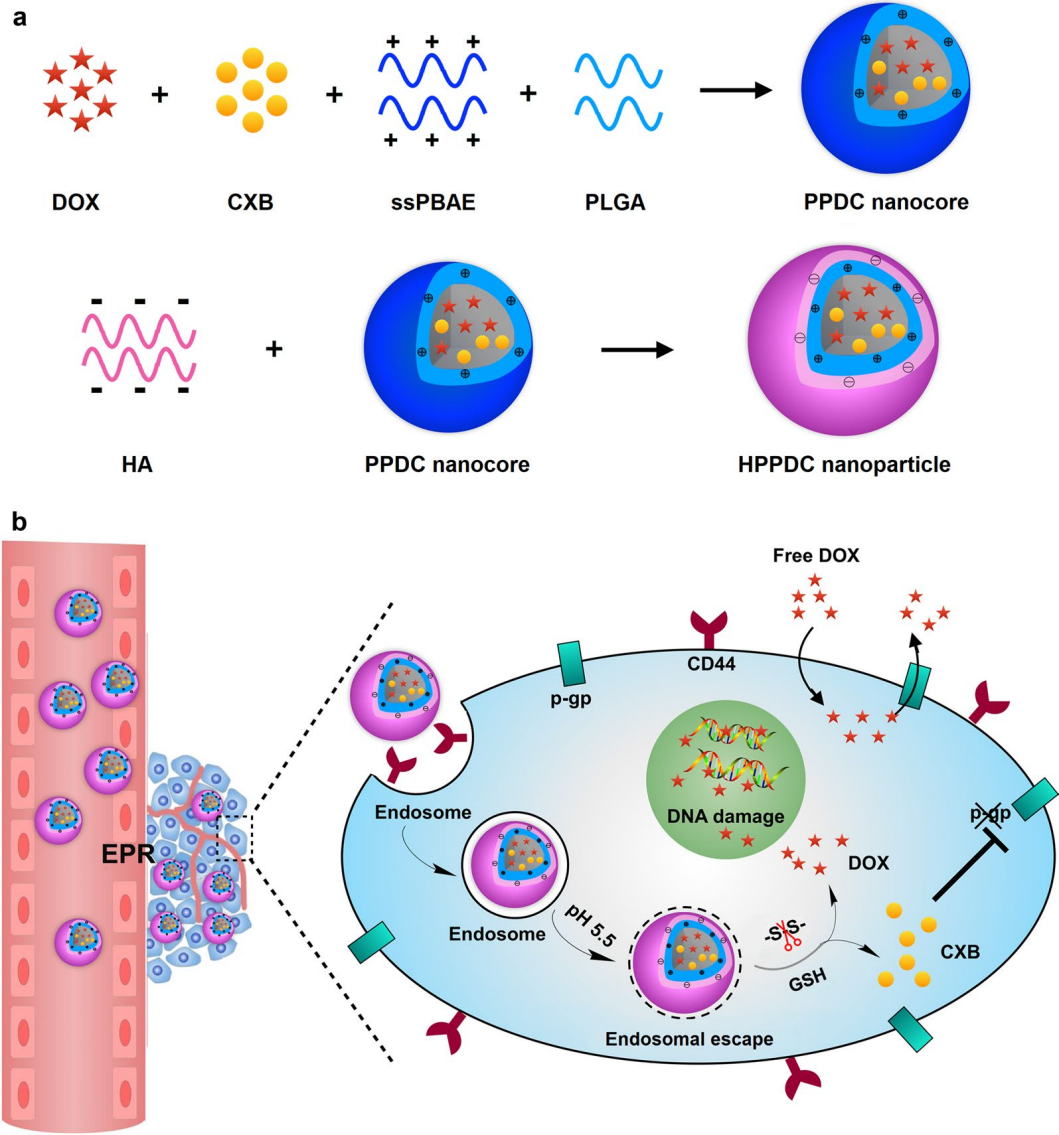
Schematic illustrations for preparation of HPPDC nanoparticles (**a**) and their functional mechanisms for overcoming drug resistance in breast cancer treatment (**b**)

In this study, we designed an intelligent nanoparticle system based on disulfide-containing poly(β-amino ester) (ssPBAE) for the co-loading, targeted delivery and controlled release of DOX and CXB. ssPBAE is a novel cationic polymer possessing pH and redox dual sensitivities due to the presence of both tertiary amine group and disulfide bond in its monomer structure and we have successfully used it as a carrier material for intracellular delivery of genes and anticancer drugs [28, 29]. Here ssPBAE is firstly complexed with poly(lactic-co-glycolic acid) (PLGA) to prepare hydrophobic nanocores containing DOX and CXB (PPDC nanocores), and then hyaluronic acid (HA) coats on these nanocores through charge interactions to form “core–shell” structured HPPDC nanoparticles (Fig. 1a). According to the previous reports [30], surface modification with HA will help to improve the hemocompatibility of hydrophobic nanoparticles, increased their in vivo stability and prolong their blood circulation time. As a natural ligand for CD44 that is often over-expressed in breast cancer cells, HA can also effectively mediate breast cancer-targeted drug delivery [31, 32]. Based on our design, HPPDC nanoparticles can exert synergistic functions against drug resistance in breast cancer over-expressing CD44 and P-gp through multiple mechanisms, which are illustrated in Fig. 1b. HPPDC nanoparticles can easily reach the tumor site through the EPR effect and then be internalized by breast cancer cells via specific endocytosis mediated by HA/CD44 binding, which will help to circumvent the efflux effect of P-gp. Next, the acidic pH of endosomes can trigger the escape of HPPDC nanoparticles from the endosome into the cytoplasm through the “proton-sponge” effect of ssPBAE. Afterwards the disulfide bonds in ssPBAE will be cleaved due to the high concentration of intracellular GSH, thus leading to the rapid degradation of HPPDC nanoparticles and burst releases of DOX and CXB. DOX will exert its cytotoxicity against breast cancer cells. CXB can selectively inhibit COX-2 and subsequently suppress the expression and function of P-gp, and therefore further enhance the chemosensitivity in breast cancer. Besides that, we also systematically investigated synergistic antitumor effects of HPPDC nanoparticles both in vitro and in vivo to evaluate their potential for overcoming drug resistance in breast cancer.

## Materials and methods

### Materials

ssPBAE was synthesized by ourselves according to the previous report [28, 29] and the polymerization degree detected by the ^1^H NMR method was about 12. HA with a molecular mass of 33 kDa was purchased from Bloomage Freda Biopharm (Jinan, China). PLGA with an average molecular weight of 20 kDa and a lactide/glycolide ratio of 50/50 was purchased from Daigang Biomaterial (Jinan, China). Celecoxib (CXB) and DOX·HCl were both obtained from Meilun Biology Technology (Dalian, China). *d*,*l*-Dithiothreitol (DTT), cyanine 5.5 (Cy5.5) and 4′,6-diamidino-2-phenylindole (DAPI) were purchased from Sigma-Aldrich (St. Louis, USA). Cell counting kit-8 (CCK-8) was obtained from Dojindo (Japan). The other chemical reagents were analytical grade from various commercial sources.

Human breast cancer MCF-7 and MCF-7/ADR cell lines, which are sensitive and resistant to DOX respectively, were both kindly gifted from the Detroit Hospital (Detroit, MI, USA). They were cultured in Dulbecco’s modified Eagle’s medium (Gibco, Life Technologies, USA) supplemented with 10% v/v fetal bovine serum (FBS) and 1% v/v penicillin/streptomycin at 37 °C in an atmosphere of 5% CO_2_. Female BALB/c nude mice were purchased from Vital River Laboratory Animal Technology (Beijing, China) and housed in a specific pathogen-free environment. The xenograft mouse model for MDR breast cancer was constructed by transplanting MCF-7/ADR cells subcutaneously to the nude mice. All of the animal experiments were carried out to the Guide for Care and Use of Laboratory Animals published by the National Institutes and the protocols were approved by the Tianjin Medical University Animal Care and Use Committee.

### Preparation and characterization of HPPDC nanoparticles

PPDC nanocores were firstly prepared using the emulsion-solvent evaporation method. Briefly, DOX·HCl was desalted in methanol containing 3 equivalents of triethylamine and afterwards vacuum dried for use. Next, different amounts of DOX were separately mixed with 600 mg ssPBAE, 200 mg PLGA and 80 mg CXB in 5 mL CHCl_3_ and continuously stirred for 6 h. These mixtures were added into 50 mL deionized water, sonicated in an ice bath for 5 min using a UH-500A ultrasonic probe (Autoscience Instrument, Tianjin, China), and then stirred at 600 rpm for 4 h to acquire PPDC nanocores with different DOX/CXB weight ratios. Then the PPDC nanocores were transferred to round bottom flask and removed CHCl_3_ by rotary evaporation in room temperature. After that, PPDC nanocores were added dropwise into 50 mL deionized water containing 0.25, 0.5, 1.0 or 1.5 g of HA respectively and further stirred for 2 h at room temperature, thus obtained HPPDC nanoparticles with different weights of HA shells. For the comparison in further experiments, HPPD nanoparticles without loading of CXB were also prepared using the same method only without adding CXB during the preparation.

PPDC nanocores and HPPDC nanoparticles were characterized using a Hitachi HT7700 transmission electron microscope (TEM, Tokyo, Japan), and their sizes, size distributions and zeta potentials were detected by a Zetasizer Nano-ZS analyzer (Malvern Instruments, UK). We also monitored size changes of PPDC nanocores and HPPDC nanoparticles during storage in deionized water and 10% FBS solution at 4 °C, thus preliminarily evaluated their stability in vitro.

### Drug loading capability and in vitro drug release of HPPDC nanoparticles

The loading contents and encapsulation efficiencies of DOX and CXB in PPDC nanocores and HPPDC nanoparticles were determined by the high performance liquid chromatography (HPLC) method, in which a HPLC system containing Waters 515 pump, Waters 2487 UV detector (Waters Technologies, Milford, MA, USA) and Waters C18 analytical column (4.6 × 250 mm, 5 μm) were used, and the column temperature was maintained at 25 °C. For detecting DOX, sample solutions were processed orderly by dilution with 0.1 M HCl solution, vibration for 3 h and filtration through a filter with 0.22 μm pore size, and afterwards injected into the HPLC system. The mobile phase consisted of acetonitrile/0.05 M KH_2_PO_4_ buffer (35/65, v/v, pH 3.0) and the detection wavelength was 480 nm. For detecting CXB, sample solutions were poured into methanol, vigorously vortexed for 1 min, centrifuged at 10,000 rpm for 30 min, and after that the supernatants were collected and then injected into the HPLC system. The mobile phase consisted of methanol/water (85/15, v/v) and the detection wavelength was 254 nm. The flow rate was set at 1 mL/min. The loading contents and encapsulation efficiencies of DOX and CXB were then calculated according to the formulas previously reported [33].

pH- and redox-responsive drug release behaviors of HPPDC nanoparticles were evaluated using the dynamic dialysis method. We investigated the in vitro releases of DOX and CXB from HPPDC nanoparticles at different pH values and with/without 10 mM DTT. Briefly, sample solutions were placed into the dialysis bags with a molecular weight cutoff of 7000 Da. At the scheduled time intervals, 2 mL of release media were collected and 2 mL of fresh release media were added meanwhile. The released amounts of DOX and CXB were then determined by the HPLC methods as described above.

### Cellular uptakes and intracellular locations of HPPDC nanoparticles

We observed the intracellular locations of HPPDC nanoparticles in MCF-7 and MCF-7/ADR cells by the confocal microscope. The cells were seeded on glass slides in the 12-well culture plates at a density of 5 × 10^4^ cells/well and cultured for 12 h. After that, the cells were incubated separately with free DOX, DOX/CXB mixture, HPPD and HPPDC nanoparticles. At 8 h and 24 h after incubation, the cells were collected, fixed with 4% paraformaldehyde, and then stained with DAPI for 10 min. Finally, all cells were mounted in DAKO mounting medium on glass slides and imaged with a FV-1000 confocal microscope (Olympus, Japan). And cellular internalizations of HPPDC nanoparticles were also compared in drug sensitive MCF-7 cells and drug resistant MCF-7/ADR cells using the flow cytometry (BD Accuri C6, USA).

### Cell viability assay

The cytotoxicities of free DOX, DOX/CXB mixture, HPPD and HPPDC nanoparticles in MCF-7 and MCF-7/ADR cells were tested using the CCK-8 assay. Briefly, the cells were seeded into the 96-well plates at a density of 5 × 10^3^ cells/well and cultured for 24 h. After that, the cells were incubated separately with free DOX, DOX/CXB mixture, HPPD and HPPDC nanoparticles at different DOX concentrations for further 48 and 72 h, respectively. The CXB concentrations for these treatments maintained at 10 μg/mL. Next, the cells were processed with the CCK-8 reagent and the absorbance in each well was measured at 450 nm using an ELX800 absorbance microplate reader (BioZtek EPOCH, Winooski, USA). The cell survival rates were calculated by comparing the absorbance values of drug-treated cells to those of untreated cells. Furthermore, the cytotoxicities of free CXB and CXB/DOX mixture in MCF-7/ADR cells were also measured at different CXB concentrations using the same method, and the DOX concentration maintained at 5 μg/mL.

### Quantitative real-time polymerase chain reaction (qPCR)

MCF-7/ADR cells were incubated separately with free DOX, free CXB, DOX/CXB mixture, HPPD and HPPDC nanoparticles for 24 h at DOX and CXB concentrations of 5 and 10 μg/mL, respectively. After that, total RNA was extracted from the cells using TRIzol^®^ reagent (Thermo Fisher Scientific, USA) and the RNA was reverse-transcribed into first-strand cDNA using the SuperScript First-Strand Synthesis System (Invitrogen). The cDNA template was amplified by qPCR using the SYBR Green PCR Master Mix (Thermo Fisher Scientific, Waltham, USA) according to the manufacturer’s protocol. The MDR1 gene expression in each sample was normalized to glyceraldehyde-3-phosphate dehydrogenase (GAPDH) expression. The primer sequences used were as follows: MDR1, forward 5′-CCC ATC ATT GCA ATA GCA GG-3′, reverse 5′-GTT CAA ACT TCT GCT CCT GA-3′; COX-2, forward 5′-TTC AAA TGA GAT TGT GGA AAA AT-3′, reverse AGA TCA TCT CTG CCT GAG TAT CTT; GAPDH, forward 5′-TGC ACC ACC AAC TGC TTA GC-3′, reverse 5′-GGC ATG GAC TGT GGT CAT GAG-3′. qPCRs were performed using a 7500 Real-Timer PCR System (Applied Biosystems, Waltham, USA) and the relative expression fold change of mRNA was calculated using the 2^−ΔΔCt^ method.

### Western blot analysis

MCF-7/ADR cells were incubated separately with free DOX, free CXB, DOX/CXB mixture, HPPD and HPPDC nanoparticles for 24 h at DOX and CXB concentrations of 5 and 10 μg/mL, respectively. Then, the cells were lysed using radioimmunoprecipitation (RIPA) assay buffer and the protein concentrations were determined by the bicinchoninic acid protein assay kit (Thermo Fisher Scientific, USA). Equal amounts of total protein were separated by SDS-PAGE, transferred onto the polyvinylidene fluoride membranes (Bio-Rad Laboratories, USA) and blocked with 5% skim milk. The membrane was subsequently incubated with primary antibodies against GAPDH, P-gp (Abcam, UK) and COX-2 (Cell Signaling Technology, USA) overnight at 4 °C, and then processed with peroxidase-conjugated anti-mouse secondary antibody (Cell Signaling Technology, USA) for 2 h. The immunoreactive bands were visualized using a Clarity™ Western ECL Blotting Substrate (Bio-Rad Laboratories, USA) and the signal was analyzed using a BandScan software (4.0, Glyko Biomedical, Hayward, CA, USA). All values were normalized to those of GAPDH.

### Cell apoptosis analysis

MCF-7 and MCF-7/ADR cells were seeded into the 6-well plates at a density of 2 × 10^5^ cells/well and cultured for 24 h. Then the culture media were removed and the fresh culture media containing free DOX, DOX/CXB mixture, HPPD and HPPDC nanoparticles were separately added at DOX concentrations of 0.5 μg/mL for MCF-7 cells and 5 μg/mL for MCF-7/ADR cells. After further incubation for 24 h, the cells were processed with the apoptosis detection kit of Annexin V-APC/7-amino-actinomycin D (7-AAD) (BD Biosciences, USA) according to the manufacturer′s instructions and finally detected using a flow cytometer.

### In vivo biodistribution and tumor accumulation of HPPDC nanoparticles

HPPDC nanoparticles were fluorescently labeled by encapsulating Cy5.5 (a near-infrared dye) into PPDC nanocores during preparation, and then their tissue distribution and tumor-accumulation in MCF-7/ADR tumor-bearing mice after administration were analyzed using the in vivo bioluminescence imaging technique, thus to evaluate their breast cancer targeting capability. Briefly, the mice were separately injected with normal saline as the control, free Cy5.5, PPDC/Cy5.5 nanocores, and HPPDC/Cy5.5 nanoparticles through the tail vein at the same Cy5.5 concentration, and then imaged using the IVIS in vivo imaging system (PerkinElmer, USA) respectively at 2, 6 and 24 h post administrations. After that, all mice were sacrificed by cervical dislocation, and their major organs (heart, liver, spleen, lung, and kidney) and tumors were collected for further fluorescence imaging.

### In vivo antitumor efficacy of HPPDC nanoparticles

MCF-7/ADR tumor-bearing nude mice were randomly divided into 7 groups with 8 mice per group and treated separately with normal saline as the control, free CXB, free DOX, DOX/CXB mixture, PPDC nanocores, HPPD and HPPDC nanoparticles. The doses of DOX and CXB were 4 and 8 mg/kg, respectively. All administrations were carried out via intravenous injection every other day for consecutive 5 times. During the whole treatment period, the tumor sizes and body weights were measured every 2 days. When the treatments were completed, all mice were sacrificed, and then the major organs and tumors were excised for further experiments.

### Histopathological examinations

The above organs and tumors were fixed in 4% formalin, dehydrated with a graded ethanol series, embedded in paraffin, and cut into 5-μm thick slices. Next, these sections were stained with hematoxylin and eosin (H&E, Sigma-Aldrich, USA) and then imaged with a IX71 fluorescence microscope (Olympus, Japan). Next, the terminal deoxynucleotidyl transferase-biotin nick end-labeling (TUNEL) assay was used to detect the apoptotic cells in tumors. Briefly, tumor sections were processed with a commercial kit (DeadEnd Colorimetric TUNEL system, Promega G7130) according to the manufacturer′s instructions and then imaged with a fluorescence microscope.

### Expressions of COX-2 and P-gp at mRNA and protein levels in tumor tissues

Total RNA was extracted from each tumor using Trizol (Invitrogen, Carlsbad, CA) according to the manufacturer′s protocol. The mRNA expression levels of MDR1 and COX-2 were detected using the qPCR method as described above. Protein was extracted from each tumor with T-PER tissue protein extraction reagent (Pierce Biotechnology, Rockford, IL, USA) and then the protein expressions of P-gp and COX-2 were analyzed using the Western blot method as above described.

### Statistical analysis

Each experiment was repeated at least three times. All of the data are expressed as mean ± standard deviation. Statistical analysis was performed using one-way analysis of variance, and *P* < 0.05 was considered to be statistically significant.

## Results

### Preparation and characterization of HPPDC nanoparticles

**Fig. 2:**
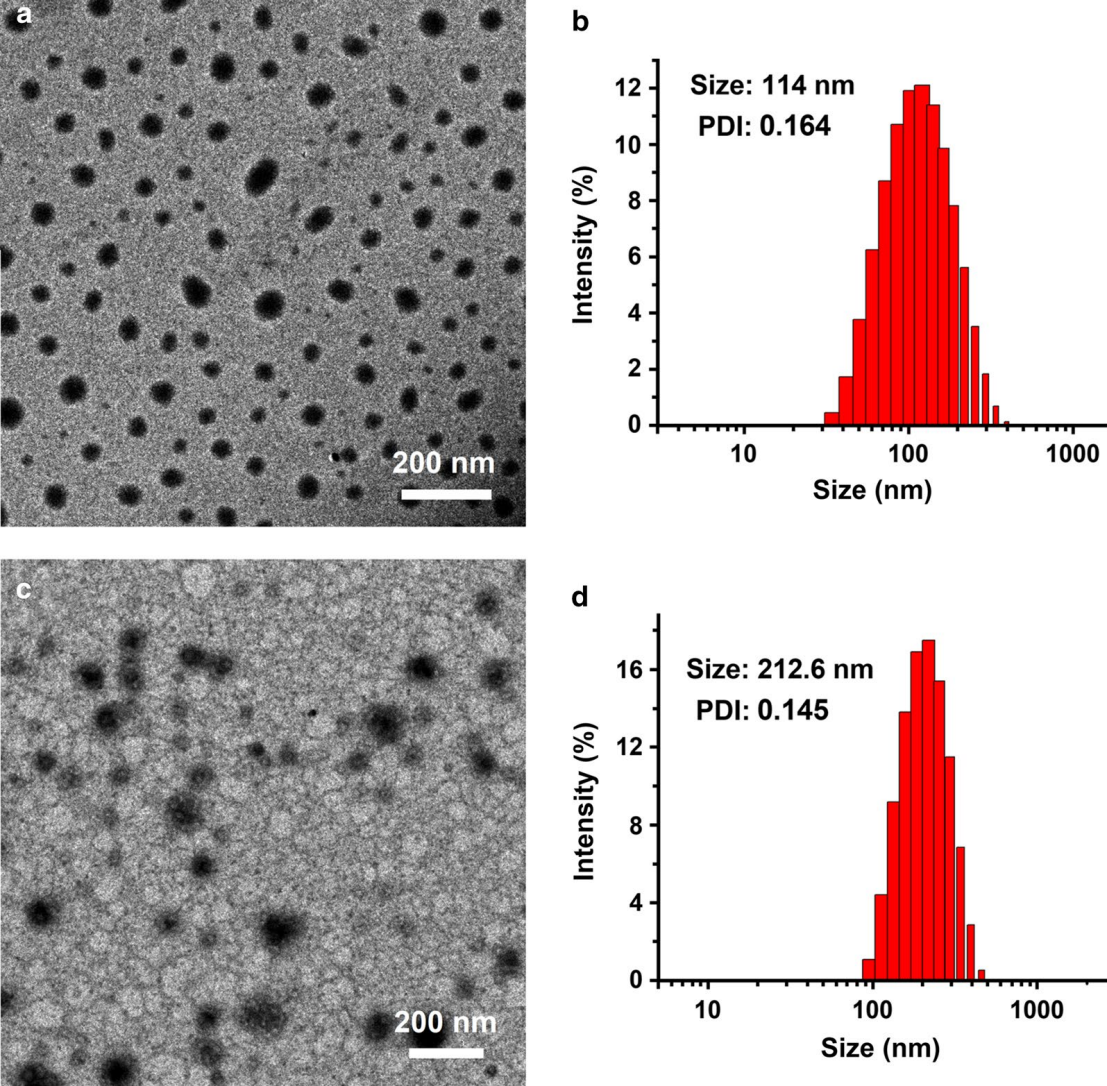
Morphological characterization of HPPDC nanoparticles. TEM images of PPDC nanocores (**a**) and HPPDC nanoparticles (**c**). Size distributions of PPDC nanocores (**b**) and HPPDC nanoparticles (**d**). The weight ratio of ssPBAE/PLGA in PPDC nancores was 3/1 and the weight ratio of HA/PPDC nanocores in HPPDC nanoaprticles was 1/1. The loading contents of DOX and CXB were 3.92% and 7.98%, respectively

In this study, a novel therapeutic system (HPPDC nanoparticles) was designed for overcoming drug resistance in breast cancer by combining chemotherapy and COX-2 inhibitor. HPPDC nanoparticles consisted of hydrophilic HA shells and hydrophobic PPDC nanocores co-loaded with a chemotherapeutic drug DOX and a COX-2 selective inhibitor CXB. Our previous investigations have shown that ssPBAE has significant amphiphilic property and can self-assemble to form nanoparticles for efficiently loading genes and/or anticancer drugs [28, 29]. Firstly, ssPBAE was synthesized and chemically characterized by ^1^H NMR spectrum (Additional file 1: Fig. S1), which was basically consistent with previously reported. Then, ssPBAE was complexed with PLGA at a weight ratio of 3/1 and used to prepare PPDC nanocores with different DOX and CXB loading contents using the emulsion-solvent evaporation method. As shown in the TEM image (Fig. 2a), PPDC nanocores had a regularly spherical shape and a compact structure, and their size determined by the dynamic light scattering method was approximately 114 nm with a relatively narrow distribution (Fig. 2b). Due to the cationic polymer nature of ssPBAE, PPDC nanoparticles exhibited a highly positively charged surface and their zeta potential was about + 38 mV.

HA is an anionic linear polysaccharide composed of alternating units of β-1,4-d-glucuronic acid-β-1,3-N-acetyl-d-glucosamine and has been widely used as a carrier material for cancer treatment because of its excellent properties such as biocompatibility, biodegradability and tumor-targeting ability. Consideration of a large amount of negative charges in its molecule, we believed that HA could be efficiently coated on the surfaces of positively charged PPDC nanocores through electronic interactions and thus to improve their in vitro and in vivo properties. Hence, we prepared HPPDC nanoparticles at different weight ratios of HA/PPDC nanocores using a simple adsorption method. The characterization parameters of HPPDC nanoparticles are shown in Additional file 1: Table S1. All HPPDC nanoparticles exhibited negative zeta potentials, confirming that HA was located on the surfaces of PPDC nanocores. As the weight ratio of HA/PPDC nanocores increased from 0.25/1 to 2/1, HPPDC nanoparticles displayed gradually increased particle size and widened size distribution. We also evaluated the stabilities of HPPDC nanoparticles in 10% FBS solution by monitoring their size changes during storage for 2 weeks at 4 °C. As shown in Additional file 1: Fig. S2, HPPDC nanoparticles at a HA/PPDC nanocores weight ratio of 1/1 had a much smaller size and higher storage stability as compared to the other HPPDC nanoparticles. Therefore, 1/1 was considered as an optimal weight ratio of HA/PPDC nanocores for preparation of HPPDC nanoparticles and used in our following experiments. HPPDC nanoparticles maintained spherical shape and showed a classic “core–shell” structure (Fig. 2c), and their size was approximately 210 nm with a relatively narrow distribution (Fig. 2d).

### Drug loading and in vitro release behaviors of HPPDC nanoparticles

In order to meet the needs of subsequent experiments, we prepared PPDC nanocores and HPPDC nanoparticles with different addition amounts of DOX and CXB, and also evaluated their loading capability for these two drugs. Our results showed that the total loading content of both DOX and CXB in PPDC nanocores could reach up to approximately 13.0% with encapsulation efficiencies more than 70%. When the addition amounts of DOX and CXB were 40 mg and 80 mg, their loading contents were 3.92% and 7.98% respectively for PPDC nanoparticles, and 2.15% and 4.02% respectively for HPPDC nanoparticles. Thus it can be seen that the coating process of HA almost could not lead to any leakage of DOX and CXB from PPDC nanocores. All above results demonstrated that both PPDC nanocores and HPPDC nanoparticles could effectively co-load DOX and CXB, thus will help to exert the synergistic anticancer effects of these two drugs.

**Fig. 3:**
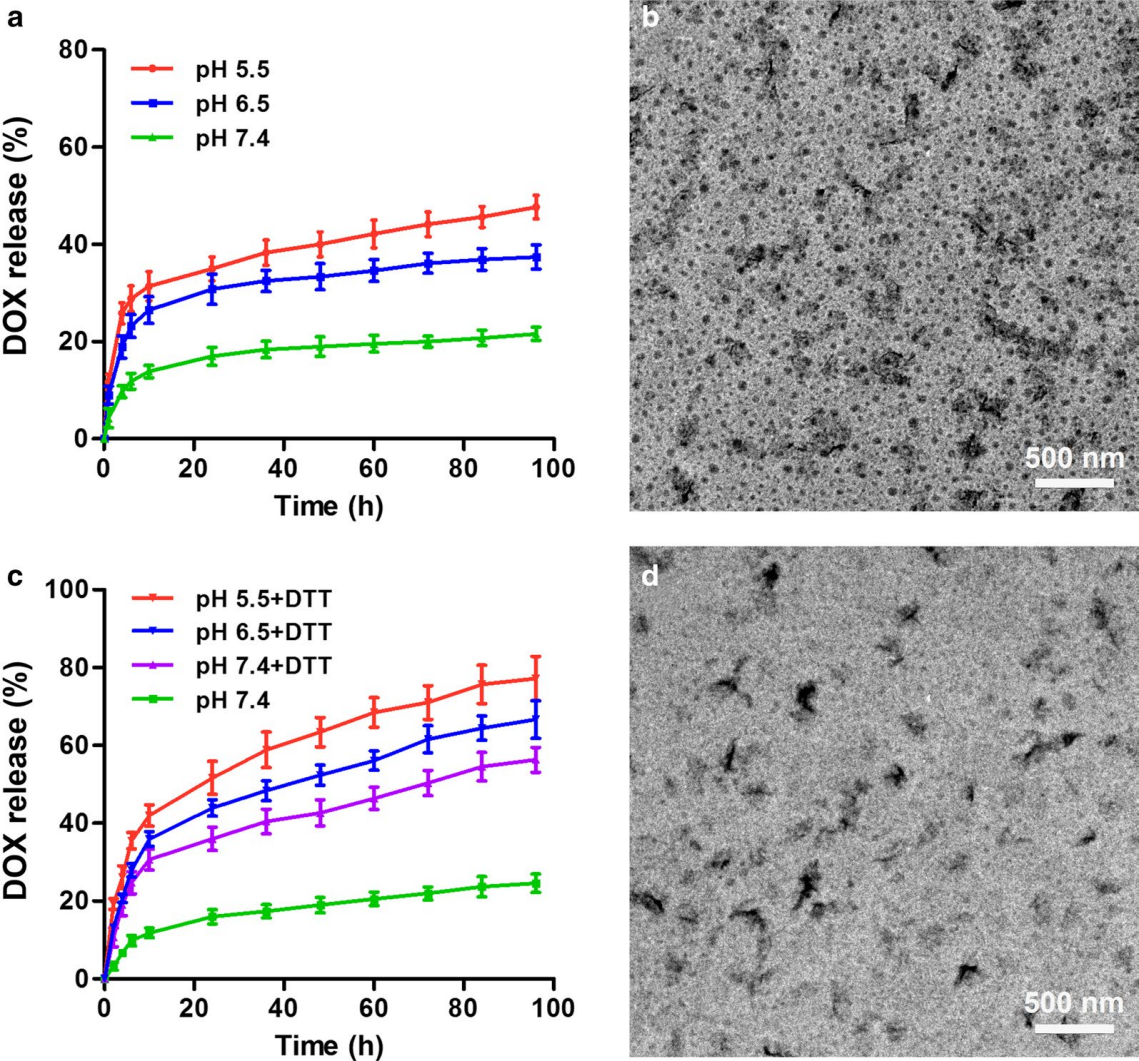
pH and redox dual-responsive drug release behaviors of HPPDC nanoparticles. **a** Release profiles of DOX from HPPDC nanoparticles at different pH values. **b** TEM image of HPPDC nanoparticles at pH 5.5. **c** Release profiles of DOX from HPPDC nanoparticles at different pH values supplemented with 10 mM DTT. **d** TEM image of HPPDC nanoparticles in PBS solution containing 10 mM DTT. Loading contents of DOX and CXB in HPPDC nanoparticles were 3.92% and 7.98%, respectively

ssPBAE exhibits pH and redox dual sensitivities due to the presence of both tertiary amine group and disulfide bond in monomer structure. Logically, we believed that HPPDC nanoparticles could controllably release drugs by responding to the acidic endo/lysosomal pH and intracellular reductive environment in cancer cells. Thus, we firstly measured the in vitro releases of DOX from HPPDC nanoparticles at pH 7.4, 6.5 and 5.5, respectively mimicking the physiological pH, early and late endosomal pH values. As shown in Fig. 3a, HPPDC nanoparticles displayed significant pH-responsive drug release profiles and the release rate was notably accelerated at both pH 6.5 and pH 5.5 as compared to that at pH 7.4. The TEM observation showed that distinct disintegration occurred in HPPDC nanoparticles in pH 5.5 release medium after drug release experiment (Fig. 3b). Next, we detected the releases of DOX from HPPDC nanoparticles in PBS solution (pH 7.4) supplemented with and without 10 mM DTT, which is often used to mimic the intracellular reductive environment (approximately 5–10 mM GSH) [34]. By contrast, the release rate of DOX from HPPDC nanoparticles was much faster in release medium containing DTT (Fig. 3c). Afterwards, these nanoparticles were thoroughly degraded and only polysaccharide aggregations were observed in their TEM image (Fig. 3d). These results demonstrated that DOX could be efficiently released from HPPDC nanoparticles in cancer cells through responding to the acidic endosomal pH and reductive intracellular environment. In addition, CXB also displayed pH- and redox dual-responsive release behaviors from HPPDC nanoparticles in vitro (Additional file 1: Fig. S3).

### Cellular uptakes and intracellular locations of HPPDC nanoparticles in drug sensitive MCF-7 cells and drug resistant MCF-7/ADR cells

P-glycoprotein (P-gp), encoded by MDR1, is an important membrane protein that can pump many foreign substances out of the cells and has been verified to be one of the major factors causing MDR in cancers. Many investigations have shown that P-gp is often over-expressed by breast cancer and may lead to chemotherapy failure by reducing the intracellular accumulation of anticancer drugs [35, 36]. Given that HA is a natural ligand for CD44, which is often over-expressed by breast cancer cells, we believed that HPPDC nanoparticles could circumvent the efflux effect of P-gp to deliver DOX and CXB through CD44-mediated endocytosis. And moreover, CXB released from HPPDC nanoparticles could down-regulate the expression of P-gp, thus further avoid the efflux of DOX from breast cancer cells. To evaluate the efficiency of HPPDC nanoparticles for overcoming P-gp mediated drug resistance, we compared their cellular uptakes and intracellular locations in drug sensitive MCF-7 cells and drug resistant MCF-7/ADR cells using the laser confocal microscope and flow cytometry. As we previously reported [37], MCF-7/ADR cells used in this study exhibit strong resistance to DOX mainly caused by P-gp and also over-express CD44 as compared to MCF-7 cells.

**Fig. 4:**
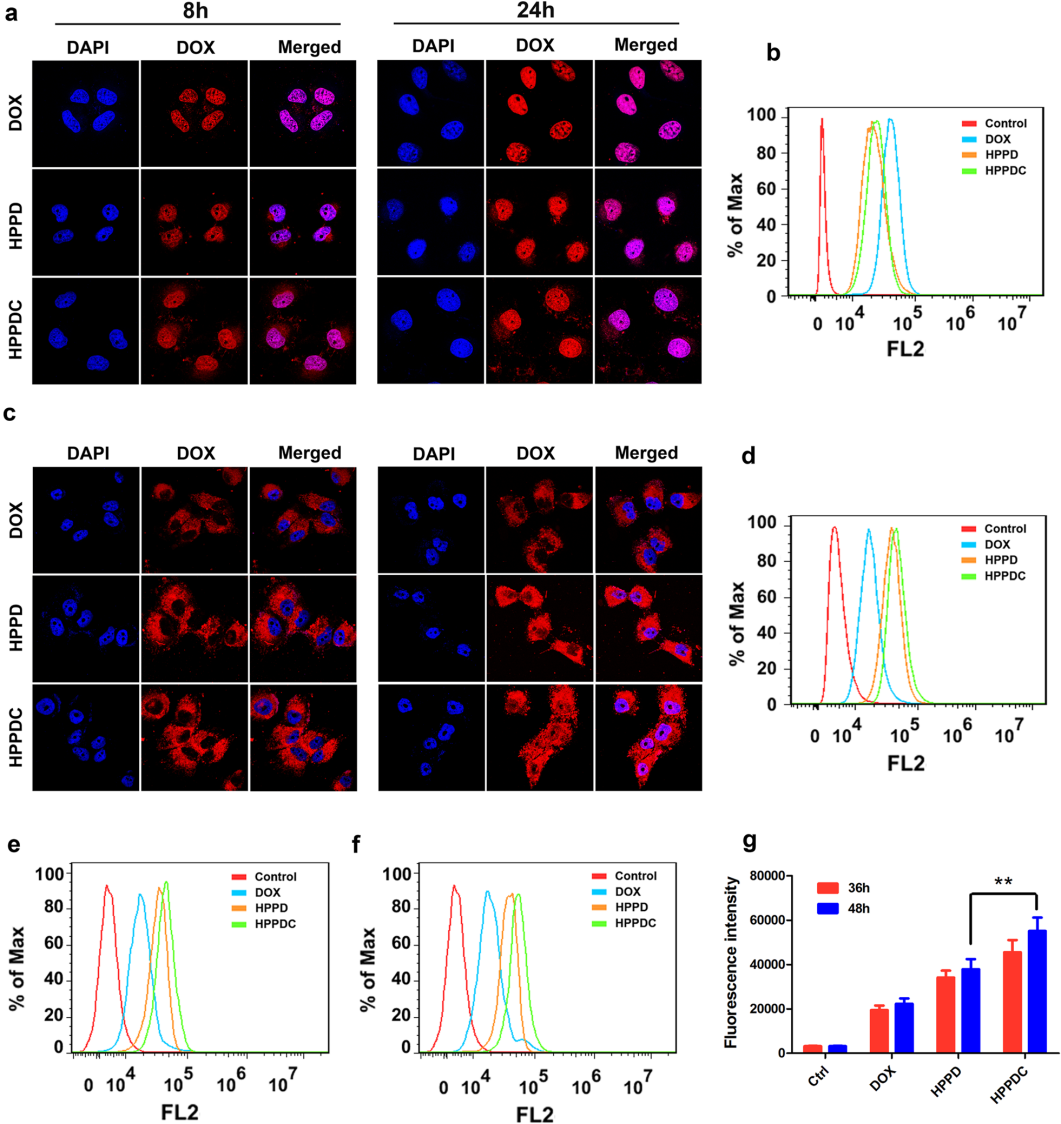
Cellular uptakes and intracellular locations of HPPDC nanoparticles. Confocal images of MCF-7 (**a**) and MCF-7/ADR cells (**c**) after incubation with free DOX, HPPD and HPPDC nanoparticles for 8 and 24 h. Flow cytometry analysis of MCF-7 (**b**) and MCF-7/ADR cells (**d**) after 24 h incubation with free DOX, HPPD and HPPDC nanoparticles. Flow cytometry analysis of MCF-7/ADR cells after 36 h (**e**) and 48 h (**f**) incubation with free DOX, HPPD and HPPDC nanoparticles. **g** The comparison of intracellular fluorescence intensities of different groups treated MCF-7/ADR cells. DOX concentration was 0.5 μg/mL for MCF-7 cells and 5 μg/mL for MCF-7/ADR cells. Cell nuclei were stained with DAPI showing a blue fluorescence. ** indicates P < 0.01 between two treatment groups

Free DOX, HPPD (without loading CXB) and HPPDC nanoparticles all successfully entered into MCF-7 cells at 8 h and 24 h after incubation, and the red fluorescence of DOX was stronger at 24 h in the cell nuclei (Fig. 4a), indicating that DOX had been released from HPPDC nanoparticles after cell entry. Compared to free DOX, HPPD and HPPDC nanoparticles both showed reduced intracellular fluorescence signals at 8 h and 24 h (Fig. 4b), which should be ascribed to the relative slower cell entry rate of these nanoparticles [38–40]. On the contrary, HPPD and HPPDC nanoparticles exhibited significantly stronger fluorescence signals in MCF-7/ADR cells than free DOX (Fig. 4c, d), demonstrating that HPPD and HPPDC nanoparticles effectively circumvented the efflux functions of P-gp. By contrast, the DOX fluorescence from HPPDC nanoparticles was slightly higher in MCF-7/ADR cells and more significantly distributed in the cell nuclei at 24 h after incubation. We further quantitatively measured cell uptake of DOX in different formulations against MCF-7/ADR cells at 36 h and 48 h. As shown in Fig. 4e–g, the uptake of HPPDC nanoparticles exhibited significantly more than HPPD nanoparticles in MCF-7/ADR cells at both 36 h and 48 h. It suggested that CXB released from HPPDC nanoparticles also exerted significant inhibitory effects on the P-gp function.

### In vitro efficiency of HPPDC nanoparticles for overcoming drug resistance

The MDR reversing capability of HPPDC was evaluated in MCF-7/ADR cells using the CCK-8 assay. We first confirmed blank nanoparticles (HPP) with high biosafety had no cytotoxicities in both MCF-7 (data not shown) and MCF-7/ADR cells (Additional file 1: Fig. S4). Free DOX exhibited a relatively low cytotoxicity (Additional file 1: Fig. S5a) and the IC_50_ value was 32.5 μg/mL in MCF-7/ADR cells at 48 h after incubation. Free CXB had no significant cytotoxicity up to 20 μg/mL (Additional file 1: Fig. S5b), but it could enhance the cytotoxicity of DOX (5 μg/mL) in a concentration-dependent manner and this chemosensitization effect tended to be gentle at the CXB concentration above 10 μg/mL (Additional file 1: Fig. S5c). Hence, 10 μg/mL was used as a treatment concentration of CXB to carry out the following experiments.

**Fig. 5:**
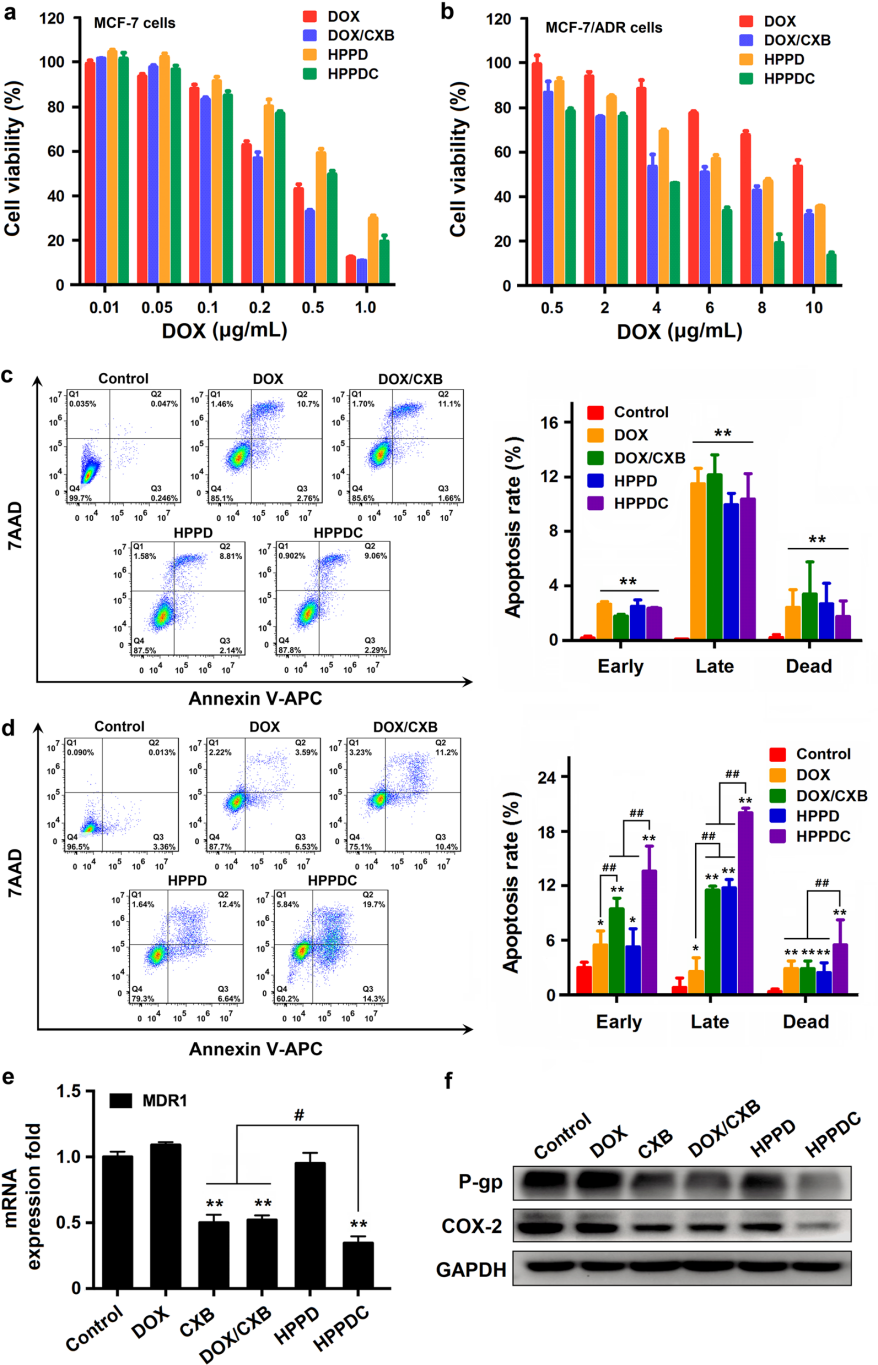
In vitro synergistic inhibitory effects of HPPDC nanoparticles on drug resistant breast cancer. Cytotoxicities of free DOX, DOX/CXB mixture, HPPD and HPPDC nanoparticles in MCF-7 (**a**) and MCF-7/ADR cells (**b**) at 72 h after incubation. Apoptosis analysis of MCF-7 (**c**) and MCF-7/ADR cells (**d**) at 24 h after various treatments (Q1: dead cells; Q2: late apoptotic cells; Q3: early apoptotic cells; Q4: living cells). **e** Relative mRNA expressions of MDR1 in MCF-7/ADR cells at 24 h after various treatments. GAPDH was used as an internal control. **f** Western blotting analyses of COX-2 and P-gp expressions in MCF-7/ADR cells at 24 h after various treatments. * and ** separately indicate P < 0.05 and < 0.01 compared to the control; ^#^ and ^##^ separately indicate P < 0.05 and < 0.01 for comparison between two treatment groups

Next, we detected the cytotoxicities of free DOX, DOX/CXB mixture, HPPD and HPPDC nanoparticles in MCF-7 and MCF-7/ADR cells after 48 and 72-h incubation. Free DOX showed significantly higher cytotoxicity than both HPPD and HPPDC nanoparticles in MCF-7 cells (Additional file 1: Fig. S6a and Fig. 5a). For example, the IC_50_ values of HPPD and HPPDC nanoparticles were respectively 0.76 and 0.58 μg/mL DOX, higher than free DOX at 72 h (0.31 μg/mL). As free DOX could easily enter MCF-7 cells through molecular diffusion and thus exerted its anticancer effects rapidly. However, the results were just the opposite in MCF-7/ADR cells (Additional file 1: Fig. S6b and Fig. 5b). At 72 h after incubation, free DOX displayed a relatively low cytotoxicity with the IC_50_ value of 13.7 μg/mL. Besides, DOX/CXB mixture and HPPD nanoparticles both enhanced the cytotoxicity of DOX, confirmed that CD44-mediated cellular internalization and COX-2 inhibition contribute to overcome drug resistance in breast cancer. More importantly, HPPDC nanoparticles had a greater cytotoxicity in MCF-7/ADR cells and their IC_50_ value was approximately 3.6 μg/mL DOX, which was much lower than those of DOX/CXB mixture (6.5 μg/mL) and HPPD nanoparticles (7.2 μg/mL). Thus it could be deduced that HPPDC nanoparticles could overcome drug resistance in breast cancer through multiple mechanisms.

We also tested the apoptosis-inducing effects of HPPDC nanoparticles in MCF-7 and MCF-7/ADR cells using the flow cytometry. As shown in Fig. 5c, MCF-7 cells treated with free DOX, DOX/CXB mixture, HPPD and HPPDC nanoparticles exhibited no significant difference in their apoptosis rates, and more than 85% of them were alive at the DOX concentration of 0.5 μg/mL after 24-h treatments. But meanwhile, DOX/CXB mixture, HPPD and HPPDC nanoparticles all exhibited notably enhanced apoptosis-inducing effects in MCF-7/ADR cells as compared to free DOX at the DOX concentration of 5 μg/mL (Fig. 5d). The DOX/CXB mixture and HPPDC nanoparticles very significantly induced both the early and late cell apoptosis, but HPPD nanoparticles exerted more potent induction effect on the late apoptosis. Furthermore, HPPDC nanoparticles exhibited much stronger induction efficacy on the cell apoptosis than both DOX/CXB mixture and HPPD nanoparticles, and the total apoptosis rate of MCF-7/ADR cells reached 37.2% after 24-h treatment. All of these results further confirmed that HPPDC nanoparticles could overcome drug resistance in breast cancer through multiple mechanisms.

Our above results confirmed its chemosensitization efficacy in MCF-7/ADR cells, and here we further provided more direct evidences of P-gp and COX-2 expressions. First, we detected the mRNA expressions of MDR1 gene encoding P-gp in MCF-7/ADR cells after various treatments for 24 h using the qPCR technique. As shown in Fig. 5e, free CXB, DOX/CXB mixture and HPPDC nanoparticles notably down-regulated the mRNA expression of MDR1. Next, we used the western blotting method to analyze the protein expressions of P-gp and COX-2 in these treated cells. As we expected, free CXB, DOX/CXB mixture and HPPDC nanoparticles remarkably reduced the protein expressions of both P-gp and COX-2 (Fig. 5f). The above results demonstrated that CXB could indeed inhibit the P-gp expression and function through suppressing COX-2. As compared with free CXB and DOX/CXB mixture, HPPDC nanoparticles exhibited evidently enhanced inhibitory effect on the mRNA and protein expressions of P-gp. We believed it was because HPPDC nanoparticles could be specifically internalized within MCF-7/ADR cells through CD44-mediated endocytosis and then rapidly degrade to release CXB by responding successively to the acidic endosomal pH and intracellular redox environment.

### In vivo biodistribution and tumor accumulation of HPPDC/Cy5.5 nanoparticles

**Fig. 6:**
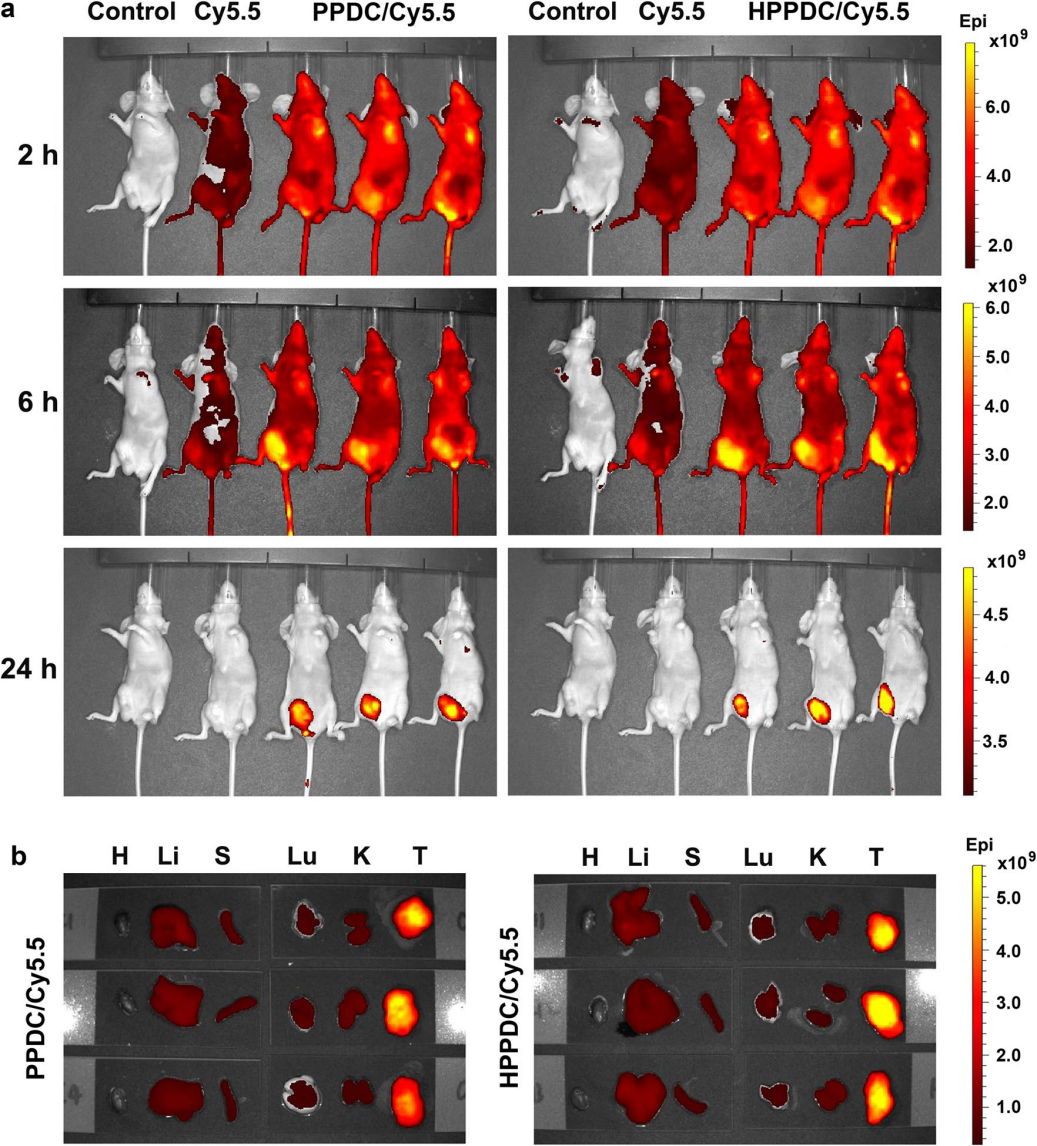
Tissue distributions and tumor accumulations in MCF-7/ADR tumor-bearing mice after intravenous injection. **a** In vivo fluorescence images of the mice at 2, 6 and 24 h after administration. **b** Fluorescence images of the major organs, including heart-H, liver-Li, spleen-S, lung-Lu, and kidney-K, and the tumors-T excised from the mice at 24 h after administration

We prepared PPDC/Cy5.5 nanocores and HPPDC/Cy5.5 nanoparticles, and then compared their tissue distributions and tumor accumulations in MCF-7/ADR tumor-bearing mice using the in vivo fluorescence imaging technique. Figure 6a shows the fluorescence images of the mice with intravenous injections of normal saline (the control), free Cy5.5, PPDC/Cy5.5 nanocores, and HPPDC/Cy5.5 nanoparticles. Free Cy5.5 was mainly located in the bladder at 2 h post injection and almost completely excreted from the mouse body at 24 h. This meant that free Cy5.5 had a rapid elimination rate in MCF-7/ADR tumor-bearing mice. The tumor distribution of free Cy5.5 was not obvious. In contrast to free Cy5.5, PPDC/Cy5.5 nanocores and HPPDC/Cy5.5 nanoparticles both showed significant tumor distributions at 6 h and merely located in the tumor at 24 h, demonstrating their excellent tumor targeting ability. We believed the EPR effect played an important role in their efficient tumor accumulation. Next, we collected the major organs including the heart, liver, spleen, lung, and kidney, and the tumors from these mice for further fluorescence imaging. As shown in Fig. 6b and Additional file 1: Fig. S7, HPPDC/Cy5.5 nanoparticles exhibited a relatively strong fluorescent signal and enhanced tumor accumulation as compared to PPDC/Cy5.5 nanocores. This should be attributed to their active targeting ability mediated by the specific binding of HA with CD44 over-expressed by MCF-7/ADR tumor. These results implied that HPPDC nanoparticles prepared in this study could be used for targeted treatment of MDR breast cancer.

### In vivo efficiency of HPPDC nanoparticles for overcoming drug resistance

**Fig. 7:**
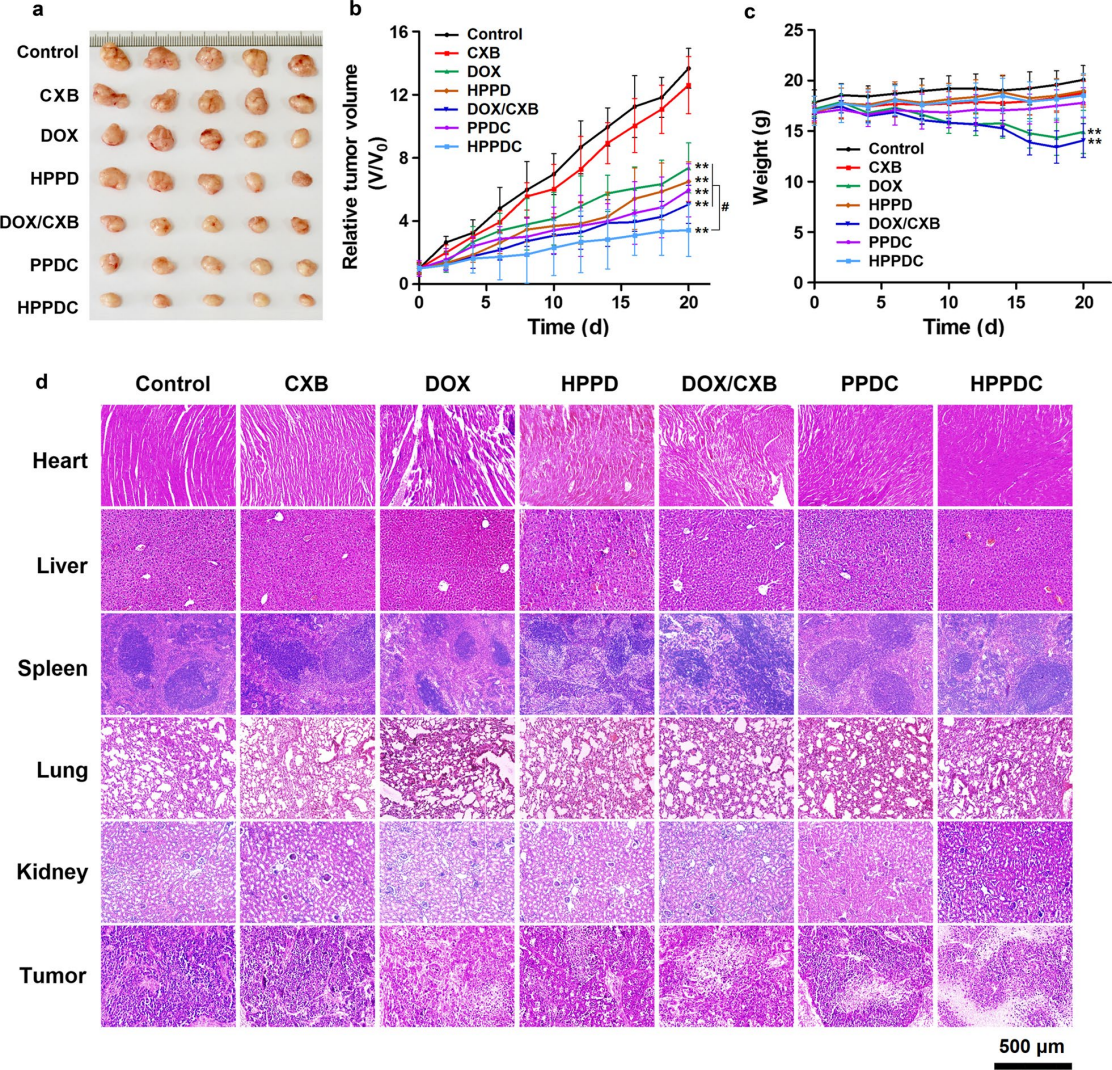
Antitumor efficacy of HPPDC nanoparticles in MCF-7/ADR tumor-bearing mice. **a** Picture of the tumors removed from the mice with treatments of normal saline (the control), free CXB, free DOX, HPPD nanoparticles, DOX/CXB mixture, PPDC nanocores, and HPPDC nanoparticles. **b** Tumor growth curves of the mice with various treatments. **c** Body weight changes in the mice with various treatments. **d** H&E stained microscopical images of the major organs and tumors removed from the mice after various treatments. ** indicates P < 0.01 compared to the control; ^#^ indicates P < 0.05 for comparison between two treatment groups

Given that HPPDC nanoparticles showed promising results as described above, we further tested their antitumor efficacy in MCF-7/ADR tumor-bearing mice. The mice were treated with normal saline (the control), free DOX, free CXB, HPPD nanoparticles, DOX/CXB mixture, PPDC nanocores, and HPPDC nanoparticles through intravenous injection every other day for consecutive 5 times. The DOX and CXB doses were 4 and 8 mg/kg, respectively. Figure 7a shows the picture of tumors removed from the mice after various treatments. It was visible that the tumors in the treatment group of HPPDC nanoparticles were smallest in size, confirming that HPPDC nanoparticles had strong antitumor efficacy in vivo. The tumor growth curves shown in Fig. 7b further confirmed it. Over the treatment course, all treatments except free CXB notably inhibited the tumor growth, but HPPDC nanoparticles also had significantly higher inhibitory effect than other treatments. Compared to both free DOX and free CXB, the DOX/CXB mixture and PPDC nanocores exhibited evidently enhanced inhibitory effect on the tumor growth, demonstrating that CXB could promote the sensitivity of drug resistant breast cancer for chemotherapy. Furthermore, HPPD nanoparticles also slightly enhanced the antitumor efficacy of DOX, which should be ascribed to their efficient tumor-accumulation through the EPR and CD44-mediated active tumor targeting. Thus it can be seen that the chemosensitization effect of CXB and tumor-targeted delivery both played important roles in the high efficiency of HPPDC nanoparticles against drug resistant breast tumor in vivo.

During the whole treatment period, we also monitored the body weight changes of the mice with various treatments and the results are shown in Fig. 7c. Free DOX and DOX/CXB mixture induced significant body weight loss in the mice due to the toxic and side effects of DOX. But in contrast, the mice with other treatments showed no significant body weight changes, demonstrating that these treatments were highly safe in their in vivo applications. We also analyzed the pathological changes of the major organs and tumors removed from these treated mice using the H&E staining. As shown in Fig. 7d, significant cardiac injury was observed in the mice treated with free DOX and DOX/CXB mixture, whereas no obvious pathological changes were visible in the heart of the mice with other treatments including PPDC nanocores, HPPD and HPPDC nanoparticles. We believed this was because that these nanoparticle formations changed tissue distributions of DOX in the mice, and thus would help to reduce its cardiotoxicity. Moreover, HPPDC nanoparticles induced more significant tumor necrosis as compared to other treatments, which should be due to their tumor-targeted drug delivery ability and efficient drug releases in breast cancer cells by responding to the acidic endosomal pH and intracellular redox environment.

**Fig. 8:**
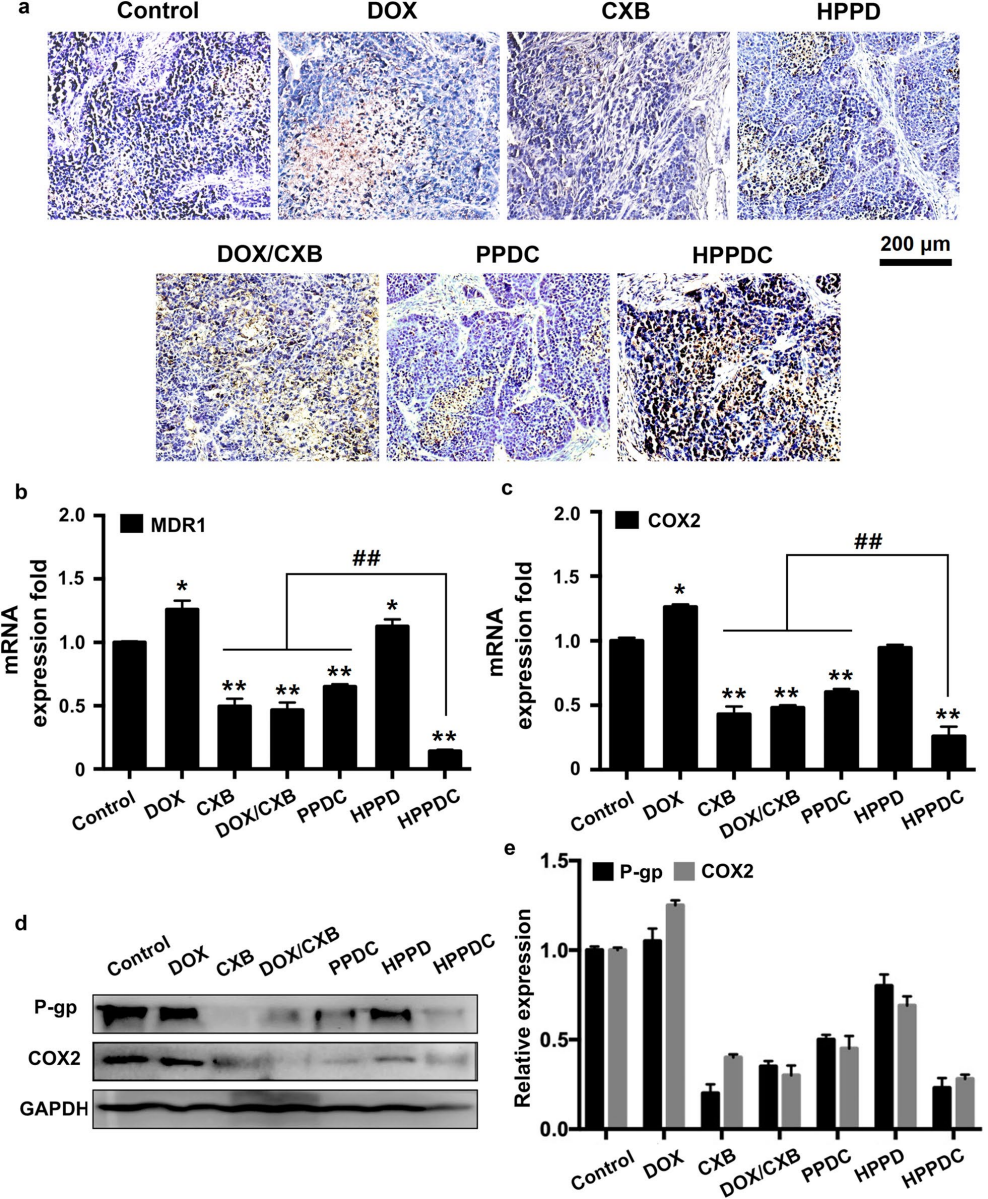
Synergistic mechanisms of HPPDC nanoparticles for overcoming drug resistance in MCF-7/ADR tumor-bearing mice. **a** Microscopical images of TUNEL-stained tumor sections from the mice with treatments of free DOX, free CXB, HPPD nanoparticles, PPDC nanocores, and HPPDC nanoparticles. Relative mRNA expressions of MDR1 (**b**) and COX-2 genes (**c**) in tumor tissues detected by the qPCR. GAPDH was used as an internal control. **d** Western blotting analyses of COX-2 and P-gp expressions in tumor tissues. **e** Quantitative comparisons of COX-2 and P-gp protein expressions in different treatment groups. * indicate P < 0.05 compared to the control; ^#^ indicates P < 0.05 for comparison between two treatment groups

We further investigated the synergistic mechanisms of HPPDC nanoparticles for overcoming drug resistance in breast cancer. First, the TUNEL staining was used to detect the apoptotic cells in the tumors after various treatments. As shown in Fig. 8a, HPPDC nanoparticles exhibited more significant effect on the induction of cell apoptosis than other treatments, confirming their strong antitumor efficacy in MCF-7/ADR tumor-bearing mice. The above results showed that MCF-7/ADR cells used in our study notably over-expressed P-gp and COX-2 (Fig. 5e, f), which should be the main reasons leading to their drug resistance. And therefore, we detected the expressions of P-gp and COX-2 at both mRNA and protein levels in the tumors. The mRNA expressions of MDR1 and COX-2 genes were firstly analyzed using the qPCR and the results are shown in Fig. 8b, c, respectively. Compared to the control, free DOX and HPPD nanoparticles enhanced the mRNA expressions of both MDR1 and COX-2, whereas free CXB, DOX/CXB mixture, PPDC nanocores, and HPPDC nanoparticles all remarkably reduced the expressions of these two genes. It indicated that CXB could reverse drug resistance in breast cancer through suppressing the P-gp and COX-2 expressions. By contrast, HPPDC nanoparticles exhibited a much higher inhibitory effect on the MDR1 and COX-2 expressions due to their efficient tumor-accumulation via the EPR effect and CD44-mediated active tumor targeting. Similar results were obtained in the protein expressions of P-gp (Fig. 8d) and COX-2 (Fig. 8e) analyzed using the Western blotting method. All these results meant that HPPDC nanoparticles had a potent reversal effect on MDR and could be used for treatment of drug resistant breast cancer by combining chemotherapy and COX-2 inhibitor.

## Conclusion

In this study, we prepared HPPDC nanoparticles composed of hydrophilic HA shells and hydrophobic ssPBAE/PLGA cores for efficient co-loading, targeted delivery and controlled release of DOX and CXB, thus hoping to acquire the synergistic effects of these two drugs against drug resistance in breast cancer. HPPDC nanoparticles showed uniform morphology, good in vitro stability, and pH/redox dual-responsive drug release behavior. At the cellular level, HPPDC nanoparticles successfully overcame drug resistant in breast cancer through multiple mechanisms including CD44-mediated cellular internalization, efficient intracellular drug release, and notably suppressed P-gp and COX-2 expressions. At the animal level, HPPDC nanoparticles exhibited excellent tumor-targeting ability and efficiently inhibited the growth of drug resistant breast tumor. In a summary, HPPDC nanoparticles show a great potential for combination treatment of drug resistant breast cancer.

## Supplementary information

**Additional file 1.** Additional table and figures.

## Data Availability

All data generated or analyzed during this study are included in this article and its additional information file.
