# Supplementary material for: pH and redox dual-responsive nanoparticles based on disulfide-containing poly(β-amino ester) for combining chemotherapy and COX-2 inhibitor to overcome drug resistance in breast cancer
Source: J Nanobiotechnology. 2019 Oct 17;17:109. doi: 10.1186/s12951-019-0540-9 (PMC6798417; doi:10.1186/s12951-019-0540-9)
Supplement: Supplementary file 1 — Additional file 1. Additional table and figures. [file 12951_2019_540_MOESM1_ESM.doc]

**Additional Material for**

**pH and redox dual-responsive** **nanoparticles** **based on disulfide-containing** **poly(β-amino ester) for** **combining chemotherapy and COX-2 inhibitor to overcome drug resistance** **in breast cancer**

Sipei Zhang^1,§^, Nan Guo^1,§^, Guoyun Wan^1^, Tao Zhang^1^, Chunyu Li^1^, Yongfei Wang^2^, Yinsong Wang^1^ (*), Yuanyuan Liu^1^ (*)

*^1^* *School of Pharmacy, Tianjin Key Laboratory on Technologies Enabling Development of Clinical Therapeutics and Diagnostics (Theranostics), Department of Genetics, School of Basic Medical Sciences, Department of Integrated Traditional Chinese and Western Medicine, International Medical School, Tianjin Medical University, Tianjin 300070, China*

*^2^ Choate Rosemary Hall, Class of 2019,* *Wallingford, Connecticut, CT 06492, USA*

🖂Corresponding author at:

Tianjin Medical University, Qixiangtai Road 22, Tianjin 300070, China

E-mail addresses:

wangyinsong@tmu.edu.cn (Yinsong Wang); liuyuanyuan01@tmu.edu.cn(Yuanyuan Liu).

**Table S1** Characteristic parameters of HPPDC nanoparticles prepared at different weight ratios of HA/PPDC nanocores

| HA/PPDC nanocores(w/w) | Size (nm) | PDI  (Polydispersity index) | Zeta (mV) |
| --- | --- | --- | --- |
| 0/1 | 115.6±2.28 | 0.182±0.034 | 38.6±0.57 |
| 0.25/1 | 172.2±3.19 | 0.149±0.038 | -21.2±1.12 |
| 0.5/1 | 203.2±4.75 | 0.141±0.012 | -24.1±1.54 |
| 1/1 | 219.1±3.91 | 0.152±0.013 | -28.1±1.24 |
| 2/1 | 304.3±3.67 | 0.16±0.053 | -32.1±0.94 |

**
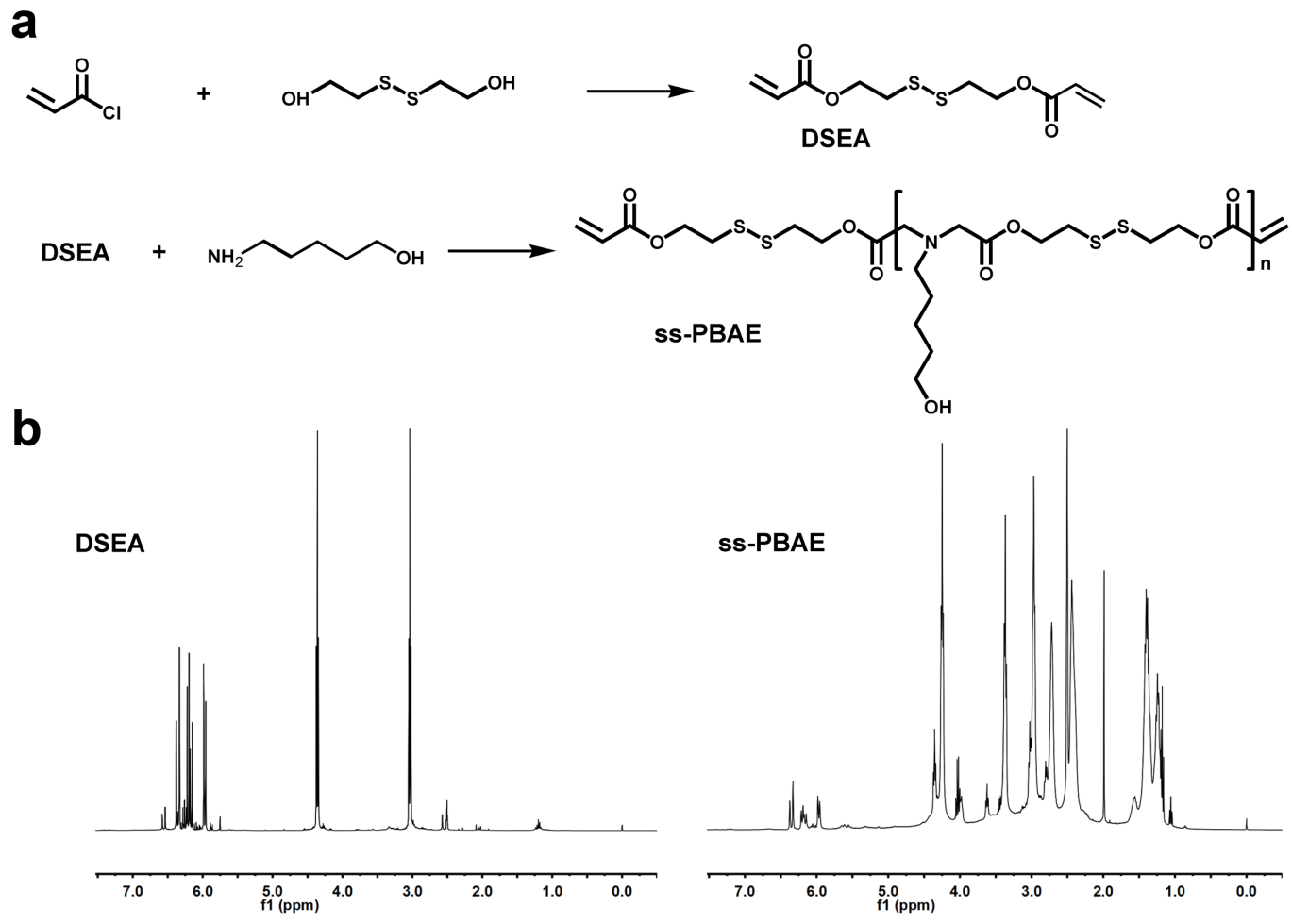
**

**Figure S1** Synthesis and characterization of DSEA and ssPBAE. The two-step reaction route of ssPBAE (a). The ^1^HNMR spectra of DSEA and ssPBAE (b).


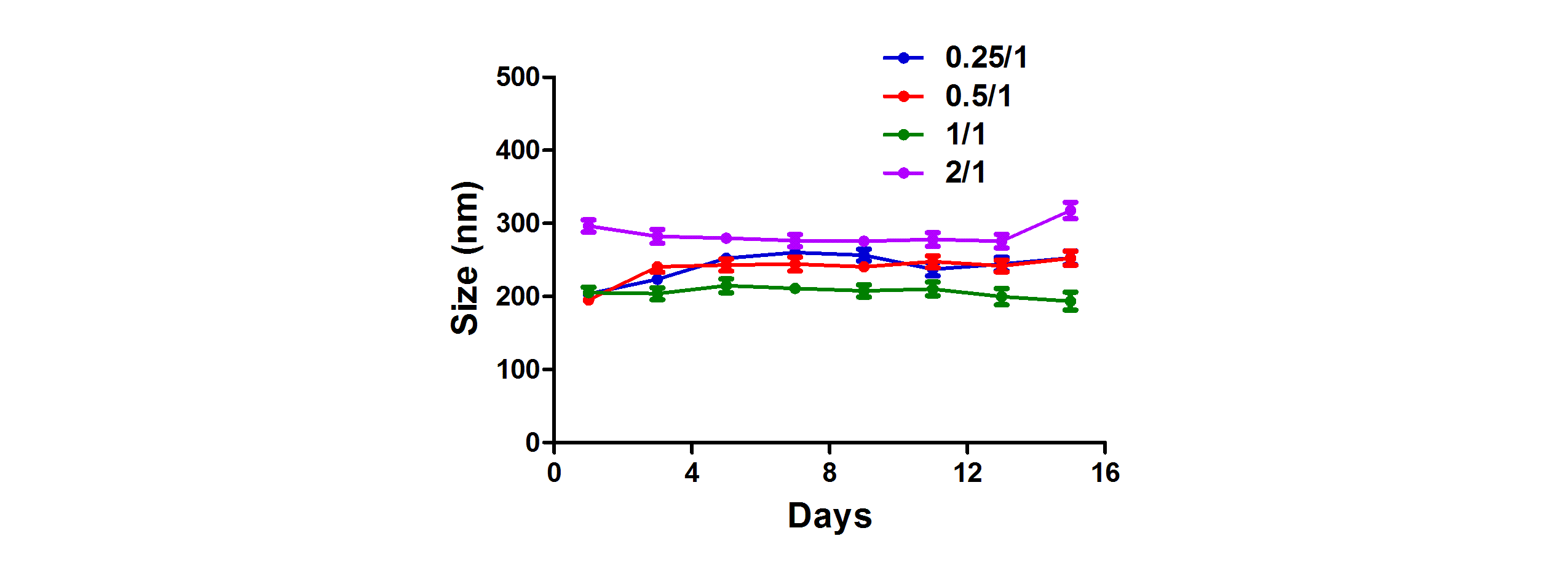


**Figure S2** Characterization of HA/ssPBAE/PLGA/DOX/CXB nanoparticles in 10 % FBS.

**
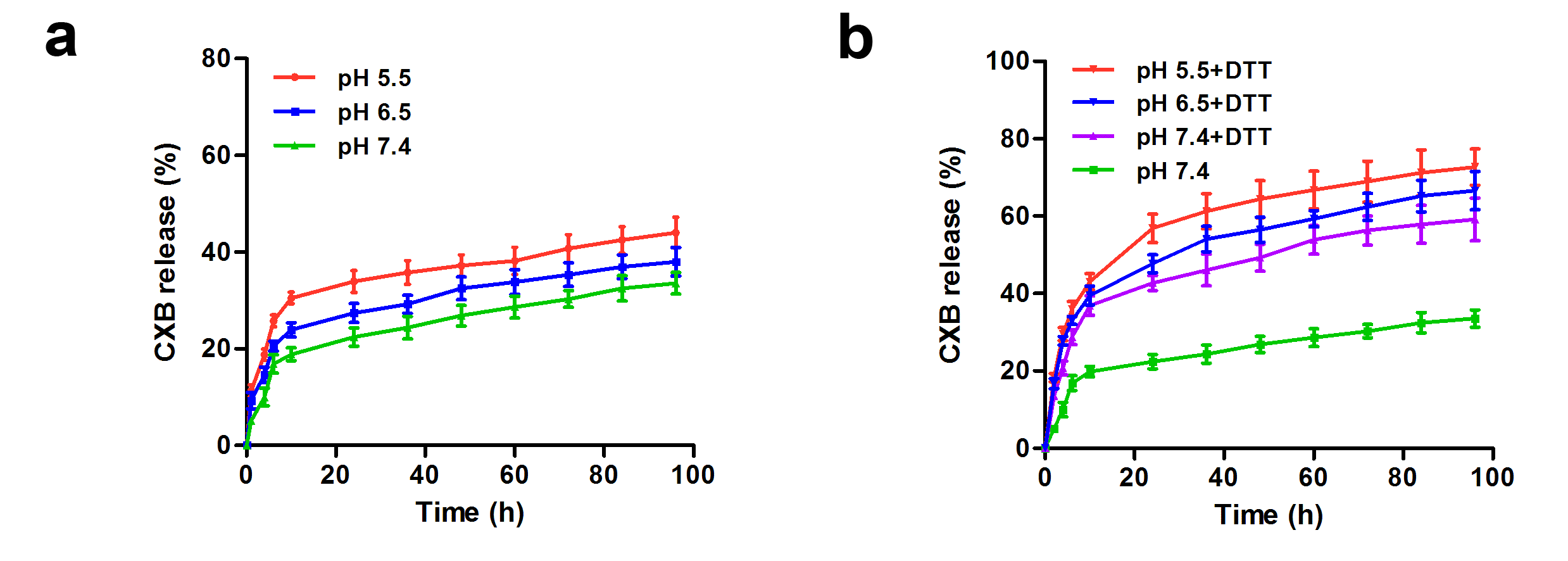
**

**Figure S3** pH and redox dual-responsive drug release behaviors of HPPDC nanoparticles. (a) Release profiles of CXB from HPPDC nanoparticles at different pH values. (b) Release profiles of CXB from HPPDC nanoparticles at different pH values supplemented with 10 mM DTT.


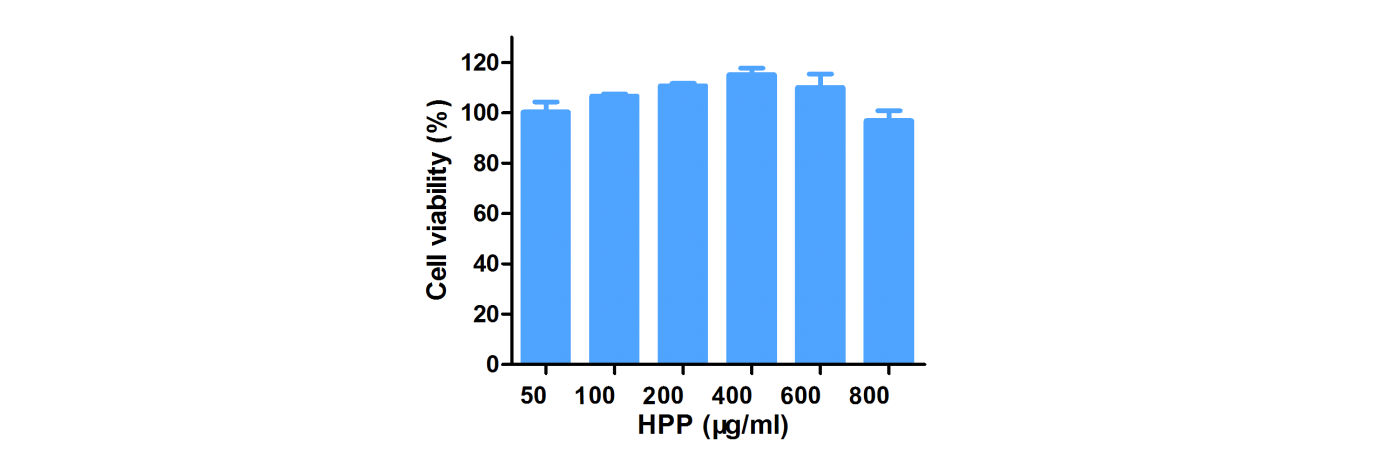


**Figure S4** Cytotoxicities of HA/ssPBAE/PLGA (HPP) nanoparticles in drug resistant MCF-7/ADR cells at different concentrations after 48-hour incubation.


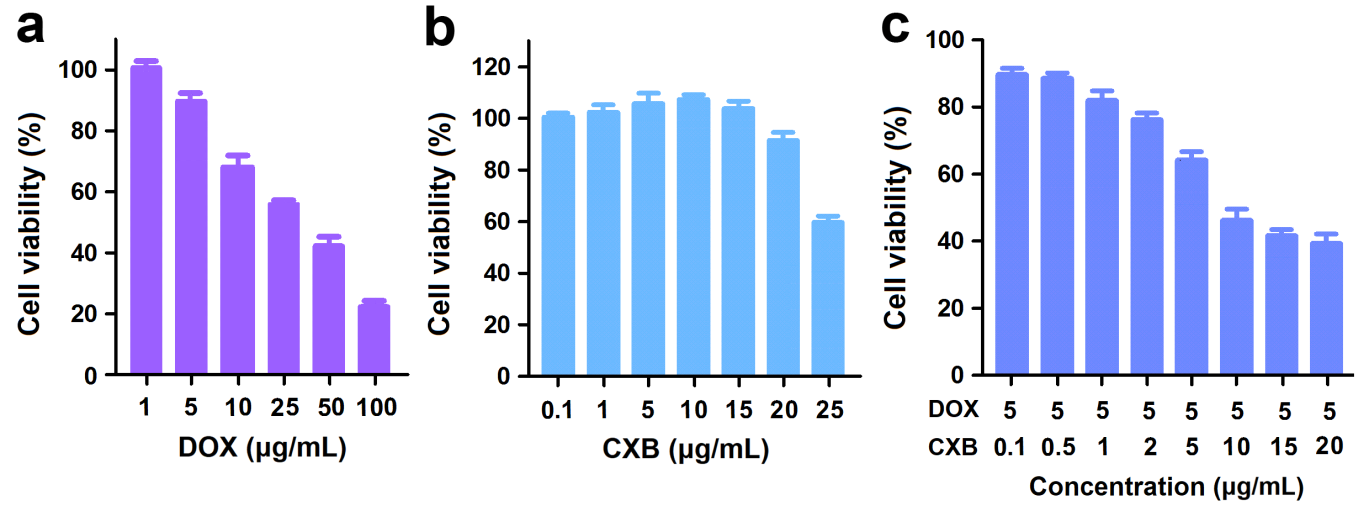


**Figure S5** Cytotoxicities of free DOX (a), free CXB (b) and DOX/CXB mixture (c) in drug resistant MCF-7/ADR cells at different drug concentrations after 48-hour incubation.


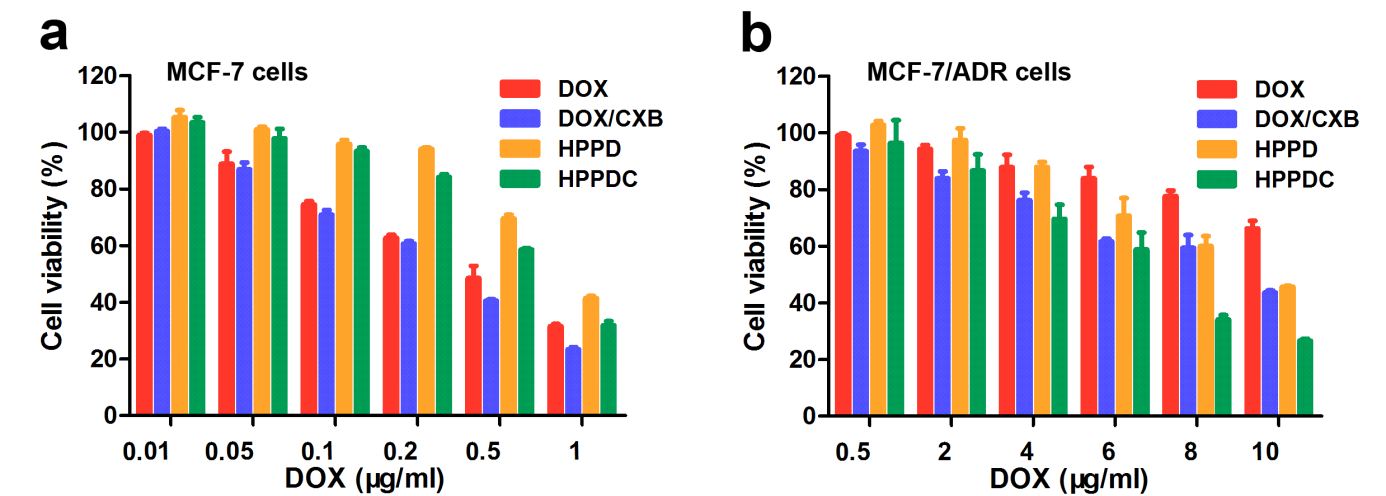


**Figure S6** Cytotoxicities of free DOX, DOX/CXB mixture, HPPD and HPPDC nanoparticles in MCF-7 (a) and MCF-7/ADR (b) cells after 48-hour incubation.


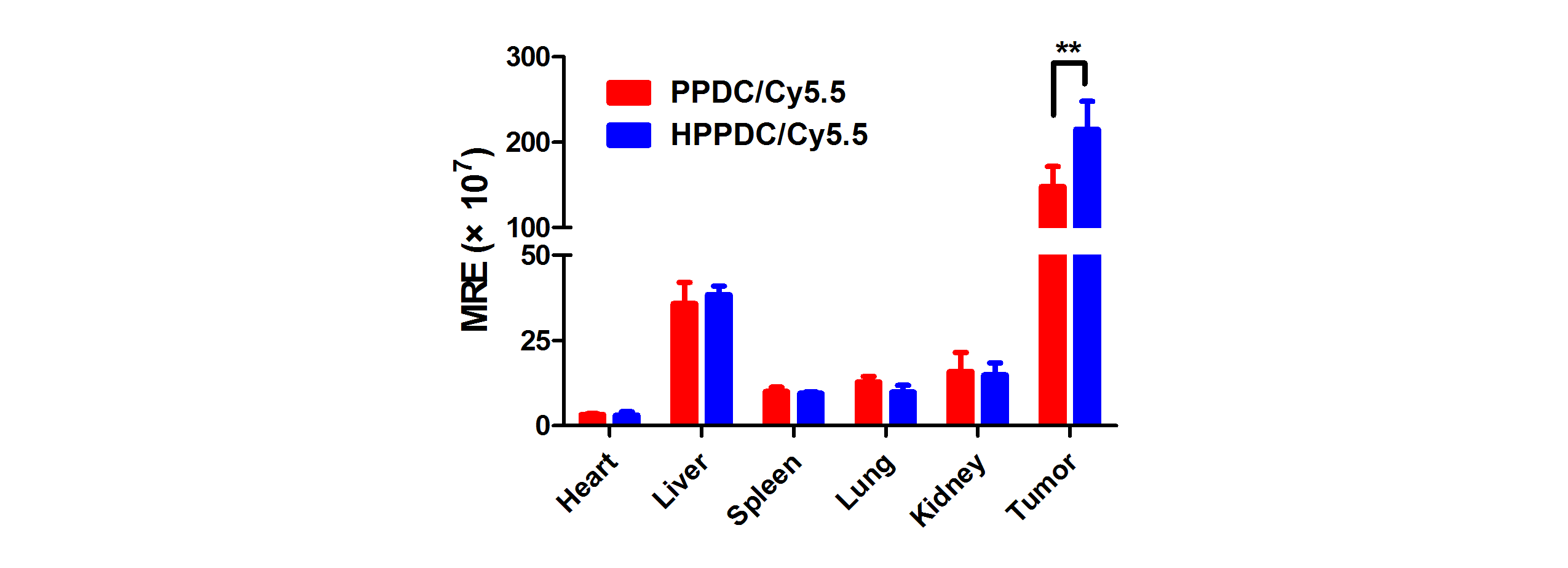


**Figure S7** Tissue distributions and tumor accumulations in MCF-7/ADR tumor-bearing mice after intravenous injection. The comparisons of mean radiant efficiencies (MREs) detected from major organs and tumor tissues at 24 h after administrations. ** indicates P <0.01 between two treatment groups.
